# Evaluating the impact of implementing an ECPR protocol on prehospital resuscitation quality: a randomized controlled simulation study

**DOI:** 10.1186/s41077-026-00458-3

**Published:** 2026-07-09

**Authors:** Frederik Marynen, Marco Garcia van Bijsterveld, Kathleen Van Loon, Sander Kempenaers, Simon Buelens, Jorg Roosen, Steffen Fieuws, Iwan C. C. van der Horst, Walther NKA van Mook, Philippe Dewolf

**Affiliations:** 1 Department of Anesthesia, University Hospitals Leuven, Herestraat 49, Leuven, 3000 Belgium; 2 Department of Emergency Medicine, University Hospitals Leuven, Herestraat 49, Leuven, 3000 Belgium; 3 Skills center KU Leuven, Herestraat 49, Leuven, 3000 Belgium; 4 Interuniversity Institute for Biostatistics and Statistical Bioinformatics, KU Leuven – University of Leuven, Leuven, 3000 Belgium; 5 Board of Directors, Albert Schweitzer hospital, Dordrecht, the Netherlands; 6 Cardiovascular Research Institute Maastricht CARIM, Maastricht University, Maastricht, the Netherlands; 7 Department of Intensive Care Medicine and Academy for Postgraduate Training, Maastricht University Medical Centre+, P. Debyelaan 25, Maastricht, 6229 HX the Netherlands; 8 School of Health Professions Education, Maastricht University, Maastricht, the Netherlands

**Keywords:** Simulation, Extracorporeal cardiopulmonary resuscitation, Advanced life support, Cardiac arrest, Resuscitation quality, Prehospital care

## Abstract

**Background:**

Extracorporeal Cardiopulmonary Resuscitation (ECPR) is increasingly considered for prehospital cardiac arrest management; however, its impact on resuscitation performance remains unclear. This study aimed to determine whether integrating an ECPR protocol into prehospital cardiac arrest care affects the quality of resuscitation compared to application of the standard Advanced Life Support (ALS) protocol.

**Methods:**

A randomized controlled simulation study was conducted at the University Hospital Leuven in Belgium using standardized pre-hospital cardiac arrest scenarios. Participants, who were physicians functioning as part of resuscitation teams, were randomized into intervention and control groups. The study included a pre- and post-intervention phase. In the pre-phase, all participants followed the standard ALS protocol. Only the intervention group received training in the additional ECPR protocol between the phases. In the post-phase, the intervention group combined this protocol with standard ALS, whereas the control group continued with ALS alone. The primary outcome was overall resuscitation quality, which was assessed using the modified Peltonen score. The secondary outcomes included occurrence and timing of critical resuscitation actions.

**Results:**

A total of 40 physicians participated in the study. Resuscitation quality was not affected by the ECPR protocol; the modified Peltonen score showed no difference in the pre-post change between the groups (0.02, CI: -0.15; 0.19, *p* = 0.83). However, secondary outcomes showed delayed actions related to the identification and management of the presumed cause of cardiac arrest in the intervention group, such as significantly later verbal suggestions to initiate causal treatments including PCI or thrombolysis.

**Conclusions:**

In this simulation study, combining a prehospital ECPR protocol with standard ALS resulted in resuscitation performance comparable to ALS alone. Nonetheless, the protocol was associated with delayed diagnostic and therapeutic actions concerning the reversible causes of cardiac arrest, highlighting the need for ECPR training that integrates diagnostic and therapeutic vigilance with procedural execution.

**Trial registration:**

Clinical Trial Center UZ Leuven, S65846 – September 2021.

**Supplementary Information:**

The online version contains supplementary material available at https://doi.org/10.1186/s41077-026-00458-3.

## Background

Out-of-hospital cardiac arrest remains a major cause of death worldwide [1]. In recent years, extracorporeal cardiopulmonary resuscitation (ECPR) has been introduced as a possible modality to support selected patients with cardiac arrest when conventional efforts fail [2–6]. Although early studies suggest that ECPR may improve outcomes, its success depends on the timely recognition of eligible patients and fast activation of the system, especially in the prehospital phase [4, 7, 8].

In contrast to some international frameworks in which prehospital cannulation is performed, the contemporary ECPR protocol in Belgium emphasizes the early identification of ECPR candidates, rapid communication with the receiving ECMO center, and expedited transport for in-hospital cannulation [4, 9, 10].

In the prehospital setting, combining an ECPR protocol with standard Advanced Life Support (ALS) means that the resuscitation team must perform more tasks in a comparable time period. These include checking ECPR’s eligibility criteria, calling the ECMO center, and preparing the patient for rapid transport, all while continuing standard resuscitation. The increased cognitive load may deflect attention from identifying and treating the reversible causes of cardiac arrest, which remain critical components of effective resuscitation [11, 12].

Although the clinical outcomes of ECPR have been studied extensively, little is known about how the use of such protocols affects the actual performance of resuscitation teams during ALS [2, 3, 7, 9]. Most studies have focused on survival or technical feasibility, rather than on how teams cope with the increased cognitive load and procedural demands introduced by ECPR protocols during the early phase of care [2–5, 7, 9, 10]. Simulation offers a valuable method to study this real-time team performance under controlled conditions [13, 14].

This study aimed to determine whether integrating a prehospital ECPR protocol into standard ALS practice affects resuscitation quality in a simulated setting. We conducted a randomized controlled simulation study comparing team leaders’ performance during resuscitation scenarios managed with standard ALS alone versus those managed with ALS in combination with an ECPR protocol, within standardized resuscitation teams.

## Methods

### Study design

**Fig. 1:**
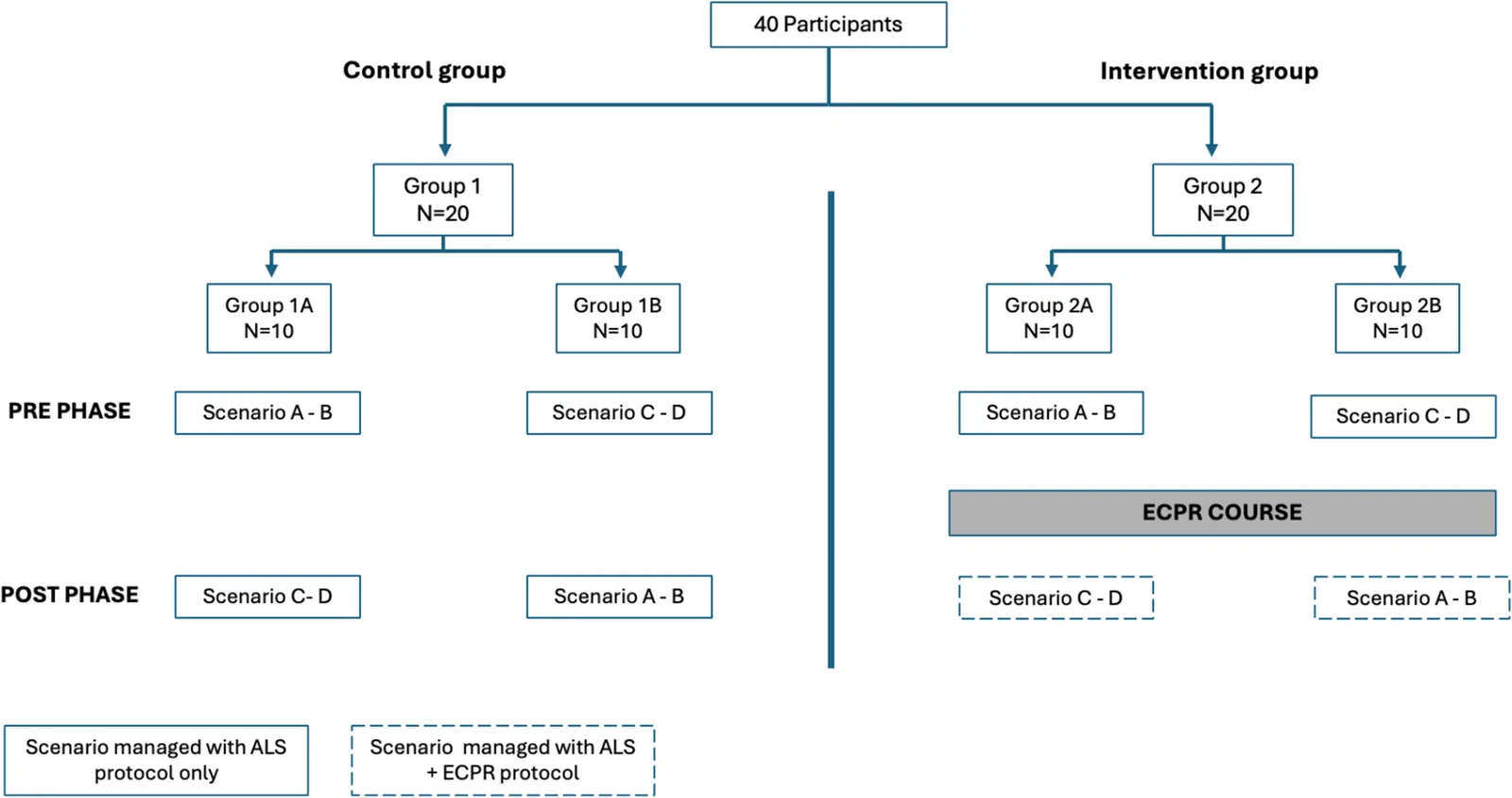
Representation of the study design showing the random assignment of participants into the control and intervention groups, the pre-and post-phase structure and the implementation of the ECPR course for the intervention group. Scenarios: A = hyperkalemia, B = intoxication, C = pulmonary embolism, D = myocardial infarction

A randomized controlled simulation study using prehospital cardiac arrest scenarios was conducted at the University Hospitals Leuven in Belgium. The study was conducted between January and June of 2022. Each participant completed two standardized cardiac arrest scenarios in a pre-phase, followed by two scenarios in a post-phase, with a minimum interval of two weeks between the phases. Participants were randomly assigned to the intervention or control group. The study design is summarized in Fig. 1.

During these simulations, participants assumed the role of team leader. The resuscitation team consisted of medical students who had been thoroughly trained to adopt standardized roles and had a solid understanding of the script. These roles represented two ambulance operators and one nurse-paramedic, aligning with the typical composition of prehospital emergency teams in Belgium.

Embedded team members (medical students) were explicitly instructed not to initiate actions independently and await direct instruction from the team leader and to execute tasks only upon request., unless predefined in the scenario script [Additional file 1].

The scenarios were standardized regarding information provided to each participant and lasted precisely 15 min. Each simulation was led by one of the six trained scenario instructors, who had been thoroughly briefed on the scenarios in advance. All the simulations were recorded for subsequent analysis.

### Simulation environment

Simulations were conducted in a dedicated Skills Lab (STEPS Skills Center KU Leuven). A prehospital environment was herein recreated to mimic real-world conditions, including typical operational constraints such as suboptimal working space. Resuscitation was performed using a Resusci Anne QCPR manikin (Laerdal, Stavanger, Norway) combined with the REALITi Go patient monitor simulator (iSimulate, Fyshwick, ACT, Australia).

### Debriefing

Following each simulation, a structured debriefing was conducted with the participant and resuscitation team, led by the scenario instructor. Two established, literature-based structured debriefing frameworks were used: DISCERN and Post-Code Pause (PCP) [15, 16]. Facilitators were trained in the application of both frameworks prior to study initiation to ensure a consistent and standardized approach to debriefing and to minimize variability between sessions.

A predefined cross-over design was used, in which each participant underwent both debriefing formats twice across the four scenarios. Exposure to both debriefing frameworks was balanced within each study phase (pre and post) and across both study groups (control and intervention), thereby minimizing systematic differences in debriefing exposure.

### Participants

Participants were physicians either in training for or had recently completed training in a recognized specialization in emergency medicine, anesthesia, or internal medicine. In addition, participants were required to have completed an ALS course within five years prior to the start of this study. This five-year timeframe was chosen to accommodate variation in ALS certification requirements across specialties during residency training in Belgium, while ensuring recent exposure to ALS principles.

The Belgian prehospital emergency response teams are typically led by a physician, supported by a nurse-paramedic and ambulance personnel. All three included specialties can serve as the prehospital physician. This study included a convenience sample of 40 participants.

### Randomisation

Using permuted blocks of size 8, 40 participants were randomly assigned to four groups (Fig. 1, Additional file 2). Two groups comprised the control group, and two comprised the intervention group. Both the control and intervention groups were divided into two subgroups to ensure that all four scenarios could be addressed in both the pre-and post-phase, thus minimizing bias. The allocation sequence was generated using a web-based randomization system accessible only by the study coordinator after the participant was enrolled [17].

### Intervention

During the pre-phase, all participants completed two simulation scenarios using the standard Advanced Life Support (ALS) protocol provided by the European Resuscitation Council (ERC) [1].

The intervention consisted of a video-based training introducing a structured ECPR protocol after the initial standard ALS resuscitation phase. The control group received no additional training.

In the training, intervention group participants were instructed to assess predefined inclusion and exclusion criteria as soon as possible after arrival on scene [Additional file 3]. If eligibility criteria were met, they were expected to contact the Emergency Department floor manager within 10 min and prepare the patient for transport while continuing resuscitation. These elements were addressed during a two-hour educational video session that included theoretical rationale, inclusion/exclusion criteria, flowchart-based activation steps, and communication with the ECMO center. The ECPR protocol trained reflects current prehospital ECPR practice in Belgium.

Before starting the post-phase, intervention group participants completed a 10-item true/false quiz on the ECPR protocol [Additional file 4]. This quiz was developed by the study team based on key elements of the ECPR protocol to verify understanding of eligibility criteria, activation procedures, and workflow steps. It was not intended to influence performance, but to serve as a formative knowledge check confirming adequate comprehension of the ECPR protocol following the training session. The quiz was not designed as a validated assessment instrument. A passing threshold of 80% (8 out of 10 correct answers) was set by internal consensus within the study team. All participants passed on their first attempt.

In the post-phase, both groups completed two previously unencountered simulation scenarios. The control group again only used the standard ALS protocol, while the intervention group applied the newly learned ECPR protocol in combination with standard ALS.

### Outcomes

Resuscitation quality in the recorded simulations as a primary outcome measure was assessed using a modified version of the ALS performance evaluation instrument originally developed by Peltonen et al. [18]. The score is based on the assessment of technical and non-technical performance during resuscitation. The instrument consists of 69 items covering seven dimensions: adherence to guidelines, clinical decision-making, workload management, team behaviour, information management, patient integrity and laymen consideration, and work routines. Items are generally scored on a 5-point scale, ranging from + 2 to − 2, with scores reflecting completely correct (+ 2), mostly correct (+ 1), not possible to evaluate due to technical failures or to other visual or audial hindrances (0), mostly incorrect (–1), or completely incorrect (–2). Some items use a simplified 3-point version of this scale, limited to + 2, 0, or − 2 [18]. The overall score per participant was calculated as the mean across all scored items, yielding a possible range of − 2.00 to + 2.00.

Because the original Peltonen instrument was developed to assess full-team performance, and because our study evaluated only the team leader while other team members fulfilled standardized non-obstructive roles, we restricted the score to items directly influenced by the team leader [Additional file 5]. Item selection was guided by a construct-driven process focusing on teamleader performance and was determined in consensus within the study team.

In the Belgian physician-led prehospital system, team composition is numerically limited, requiring the physician team leader to assume both supervisory and hands-on responsibilities during resuscitation. In addition to coordinating the team and maintaining situational oversight, the physician typically positions themselves at the head of the patient and actively manages airway and ventilation. Consequently, assessment items related to ventilation performance, as well as other airway and breathing components of the ABCDE approach, were retained.

Furthermore, embedded team members were instructed to perform standardized, non-obstructive roles and to await explicit direction from the team leader before initiating or modifying tasks. As a result, organizational aspects of resuscitation, such as rotation of chest compressors or task allocation, were dependent on team leader initiative. Items reflecting these organizational responsibilities were therefore retained, whereas items purely reflecting technical execution by team members (e.g., correct placement of defibrillator pads) were excluded.

This selection process resulted in a 41-item subset, which was used as the modified Peltonen score in the present study. Although this subset has not undergone formal psychometric validation as a standalone instrument, it preserves the theoretical structure of the original version and was specifically designed to measure team leader performance within the context of this study.

As secondary outcomes, we examined the occurrence and timing of critical resuscitation actions, some of which were scenario-specific due to the unique clinical context and underlying diagnosis. These key actions were expert-selected in consultation with the study team, and assessed through review of the video recordings of each simulation [Additional file 6].

For participants in the intervention group, adherence to and potential deviations from the newly introduced ECPR protocol were additionally evaluated. The ECPR-specific performance items were predefined by the study team prior to data analysis based on the content of the training session. The evaluated components included: (1) verification of ECPR inclusion criteria, (2) timely notification of the receiving ECPR center upon protocol activation, (3) appropriate adaptation of ALS management in accordance with the ECPR protocol, and (4) preparation of the patient for prompt transport.

### Assessment procedure

The recorded simulations were assessed by four raters, with each rater evaluating an equal share of randomly assigned simulations. All raters had extensive experience in ALS.

Prior to data collection, all raters participated in a joint training session in which they collectively reviewed and scored several example simulations. Discrepancies were discussed to align interpretation and scoring practices. Because each simulation was assessed by a single rater, no formal interrater reliability analysis was possible. Yet consistency was promoted through standardized use of the scoring tool, structured discussion, and regular consensus meetings throughout the rating process. Any uncertainties encountered during scoring were resolved through group discussion, and ratings were finalized only after full agreement was reached.

### Statistical analysis

#### Modified Peltonen score

To evaluate the Peltonen scores, a multivariate model with an unstructured covariance matrix for the four measurements per participant was fitted using the direct likelihood method to handle potential missing values for the scores. The model included the following fixed effects: group (intervention vs. control), time (pre vs. post), the interaction between group and time, discipline (3 levels), and scenario (4 levels).

The intervention effect reflects the between-group comparison of the pre-post change, the latter calculated as a linear combination of the four mean values (2 means pre and 2 means post). Results are presented using 95% confidence intervals and p-values, with the understanding that the study was exploratory in nature and not powered to test for non-inferiority based on a predefined definition of a negligible difference. A formal power analysis was not conducted, and the study used a convenience sample of 40 participants. Based on the observed standard deviation of the change in the modified Peltonen score in our data, there was 73.4% power to detect a difference of 0.20 in change between both groups (two-sided test, α = 0.05).

#### Critical resuscitation actions

For binary items, the results were reported from absolute risk models, using the same fixed-effects structure as for the Peltonen scores. Predicted probabilities (and 95% CI) from this model were reported, as well as risk differences (with 95%CI) for the pre-post change within each group and for the difference in change between both groups. Due to the low number of observations in some cells, correcting for potential correlations among the four measurements per participant was not possible. Therefore, the 95% CI and p-values should be interpreted with caution. Kaplan-Meier estimates were reported for timing variables, censoring participants who failed at 15 min. Furthermore, hazard ratios were obtained from a Cox regression model using the same fixed-effects structure as for the Peltonen scores. Robust standard errors were used to correct for the correlation between the repeated measures per participant.

All analyses were performed using SAS software, version 9.4 of the SAS System for Windows.

### Ethics approval

The Ethics Committee Research of University Hospitals Leuven approved the study on December 14, 2021 (reference number S65846). Before the commencement of the study, all participants provided signed informed consent.

### Language editing

Large Language Modelling (ChatGPT, OpenAI) was used in the preparation of this manuscript solely to improve readability and sentence structure, as the authors are not native English speakers. The authors reviewed and edited the text following this process and take full responsibility for the manuscript’s content.

## Results

### Participants and data exclusions

Forty participants were initially recruited for this study. One participant completed only the pre-phase. Additionally, technical issues during the pre-phase led to the loss of video recordings for two participants, resulting in no usable data for them. Therefore, these two participants were excluded and replaced by two newly recruited participants. These replacements were randomly assigned to the position of an excluded participant.

As a result, 40 participants had complete and usable recordings, yielding 158 simulations for analysis. The composition of the study groups is presented in Table 1.

**Table 1:** Composition of the study groups

Specialty	Level	Control	Intervention
Anesthesia	Junior Doctors	3	3
	Senior Doctors	6	8
Emergency Medicine	Junior Doctors	2	1
	Senior Doctors	4	6
Internal Medicine	Junior Doctors	1	0
	Senior Doctors	4	2
TOTAL		20	20

Junior Doctors: Physicians with ≤ 3 years of clinical experience as Medical Doctor

Senior Doctors: Physicians with > 3 years of clinical experience as Medical Doctor

Two participants who dropped out because of technical failure were excluded from this table

During some simulations, minor deviations from the scenario script were observed. Any affected data by these deviations were excluded from the analysis, and a detailed list of excluded items is provided in Additional file 7.

### Primary outcome: Modified Peltonen score (Table 2)

**Fig. 2:**
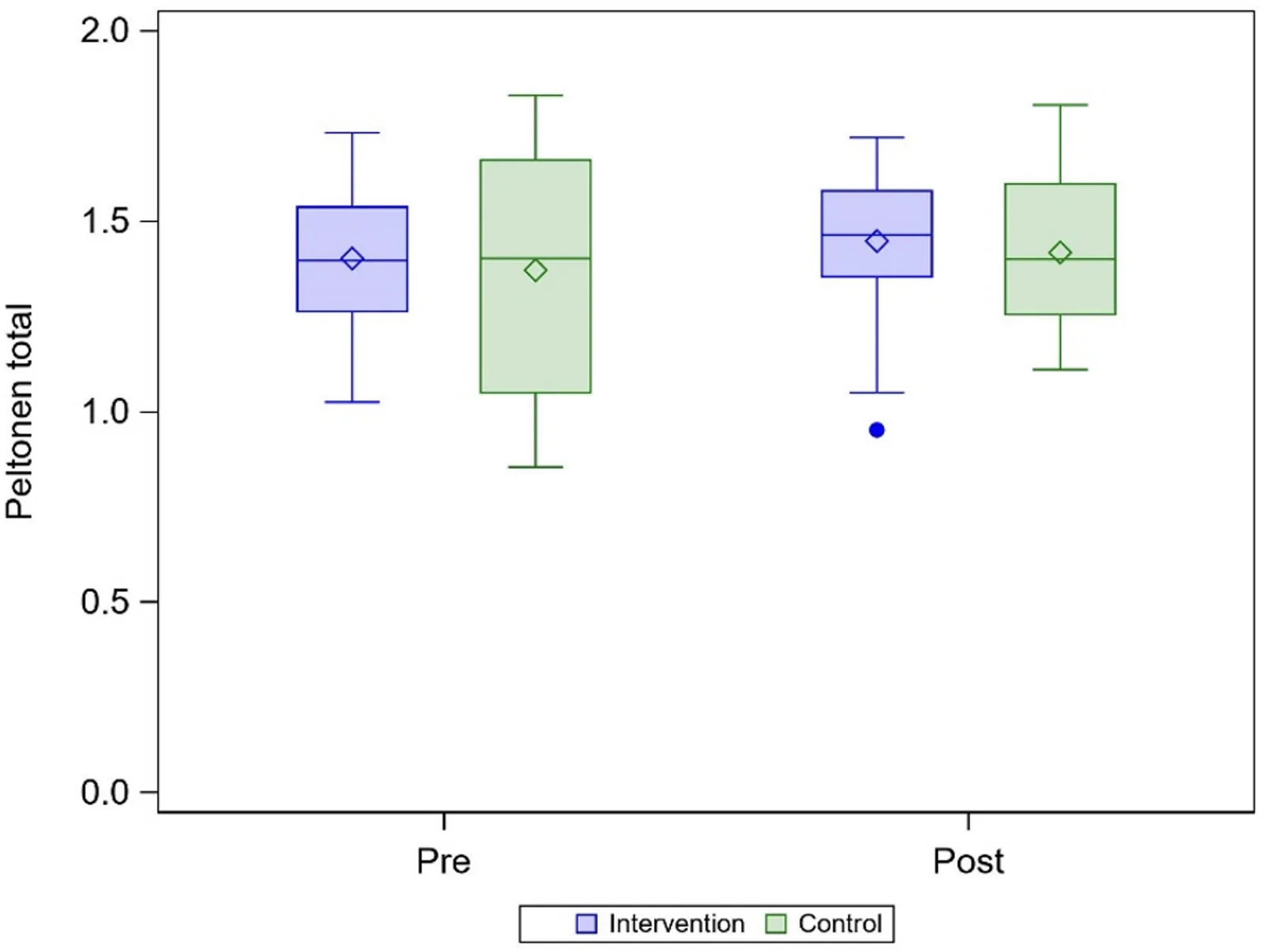
Boxplot Modified Peltonen score. The box refers to percentiles 25 and 75 (interquartile range (IQR)), with the median indicated by the horizontal line. Outliers (more than 1.5 times the IQR below or above the box) are represented by a dot. Scores range from − 2.00 to + 2.00, with higher values indicating better resuscitation performance

Regarding the total modified Peltonen score, no significant difference was observed between the pre- and post-phase in both the control (0.03, CI: -0.09 to 0.15, *p* = 0.60) and intervention group (0.05, CI: -0.07 to 0.17, *p* = 0.40). The difference in pre-post change between the control and intervention groups, which is the primary focus of this study, was 0.02 (CI: -0.15 to 0.19, *p* = 0.83). A visual representation of these findings is shown in Fig. 2.

Similarly, no significant between-group differences in pre–post change were observed for technical skills (-0.04, CI: -0.20 to 0.13, *p* = 0.68) or non-technical skills (0.05, CI: -0.17 to 0.28, *p* = 0.63).

Across all analyzed scenarios (158 simulations × 41 items), only three items were scored as 0 (“not possible to evaluate”).

**Table 2:** Results of modified Peltonen score analysis

ITEM modified Peltonen score	CONTROL			INTERVENTION			COMPARISON	
	Est. (95%CI) PRE-phase	Est. (95%CI) POST-phase	Change (95%CI)	Est. (95%CI) PRE-phase	Est. (95%CI) POST-phase	Change (95%CI)	Difference in change (95%CI)	*p*-value
TOTAL mod Peltonen score	1.39 (1.28;1.51)	1.42 (1.34;1.51)	0.03 (-0.09;0.15)	1.40 (1.28;1.52)	1.45 (1.36;1.54)	0.05 (-0.07;0.17)	0.02 (-0.15;0.19)	0.83
Technical Skills score	1.33 (1.21;1.46)	1.36 (1.28;1.44)	0.03 (-0.09;0.14)	1.45 (1.32;1.58)	1.44 (1.36;1.53)	-0.01 (-0.12;0.11)	-0.04 (-0.20;0.13)	0.68
Non-technical Skills score	1.45 (1.31;1.59)	1.50 (1.39;1.60)	0.04 (-0.11;0.20)	1.37 (1.22;1.51)	1.46 (1.35;1.57)	0.10 (-0.06;0.25)	0.05 (-0.17;0.28)	0.63
Adherence to guidelines	1.33 (1.21;1.46)	1.36 (1.28;1.44)	0.03 (-0.09;0.14)	1.45 (1.32;1.58)	1.44 (1.36;1.53)	-0.01 (-0.12;0.11)	-0.04 (-0.20;0.13)	0.68
Clinical decision making	0.98 (0.62;1.34)	1.18 (0.86;1.49)	0.20 (-0.22;0.61)	0.73 (0.36;1.11)	1.05 (0.73;1.37)	0.32 (-0.09;0.73)	0.12 (-0.46;0.70)	0.68
Workload management	1.55 (1.37;1.72)	1.60 (1.49;1.72)	0.06 (-0.14;0.25)	1.57 (1.39;1.75)	1.72 (1.60;1.84)	0.15 (-0.04;0.34)	0.10 (-0.17;0.37)	0.48
Team behaviour	1.82 (1.67;1.98)	1.84 (1.67;2.02)	0.02 (-0.18;0.21)	1.87 (1.71;2.03)	1.77 (1.59;1.94)	-0.10 (-0.29;0.09)	-0.12 (-0.39;0.16)	0.39
Information management	1.40 (1.21;1.59)	1.36 (1.22;1.51)	-0.04 (-0.23;0.15)	1.44 (1.24;1.64)	1.39 (1.24;1.55)	-0.05 (-0.24;0.14)	-0.01 (-0.29;0.26)	0.92
Patient integrity and consideration of laymen	1.80 (1.35;2.25)	1.48 (0.96;2.00)	-0.32 (-1.06;0.42)	1.22 (0.76;1.69)	1.21 (0.70;1.73)	-0.01 (-0.74;0.72)	0.31 (-0.74;1.37)	0.56
Work routines	1.14 (0.73;1.54)	1.27 (0.72;1.81)	0.13 (-0.51;0.77)	0.94 (0.50;1.37)	1.20 (0.66;1.75)	0.27 (-0.36;0.90)	0.14 (-0.77;1.05)	0.76

Least-squares estimates (with 95% confidence intervals) from the linear models for the mean in the pre- and post-phase, including estimates for the within-group pre–post change and the between-group difference in change. Est: estimate

### Secondary outcome: critical resuscitation actions (Table 3, Additional file 6)

#### Scenario-independent items

The analysis of critical resuscitation actions, unrelated to specific scenarios, generally revealed no significant between-group pre-post change difference. However, the item ‘Timing of application of capnography’ showed a significant pre-post change difference favoring the intervention group (Hazard Ratio 1.90, CI: 1.15 to 3.16, *p* = 0.01).

#### Scenario-specific items

**Fig. 3:**
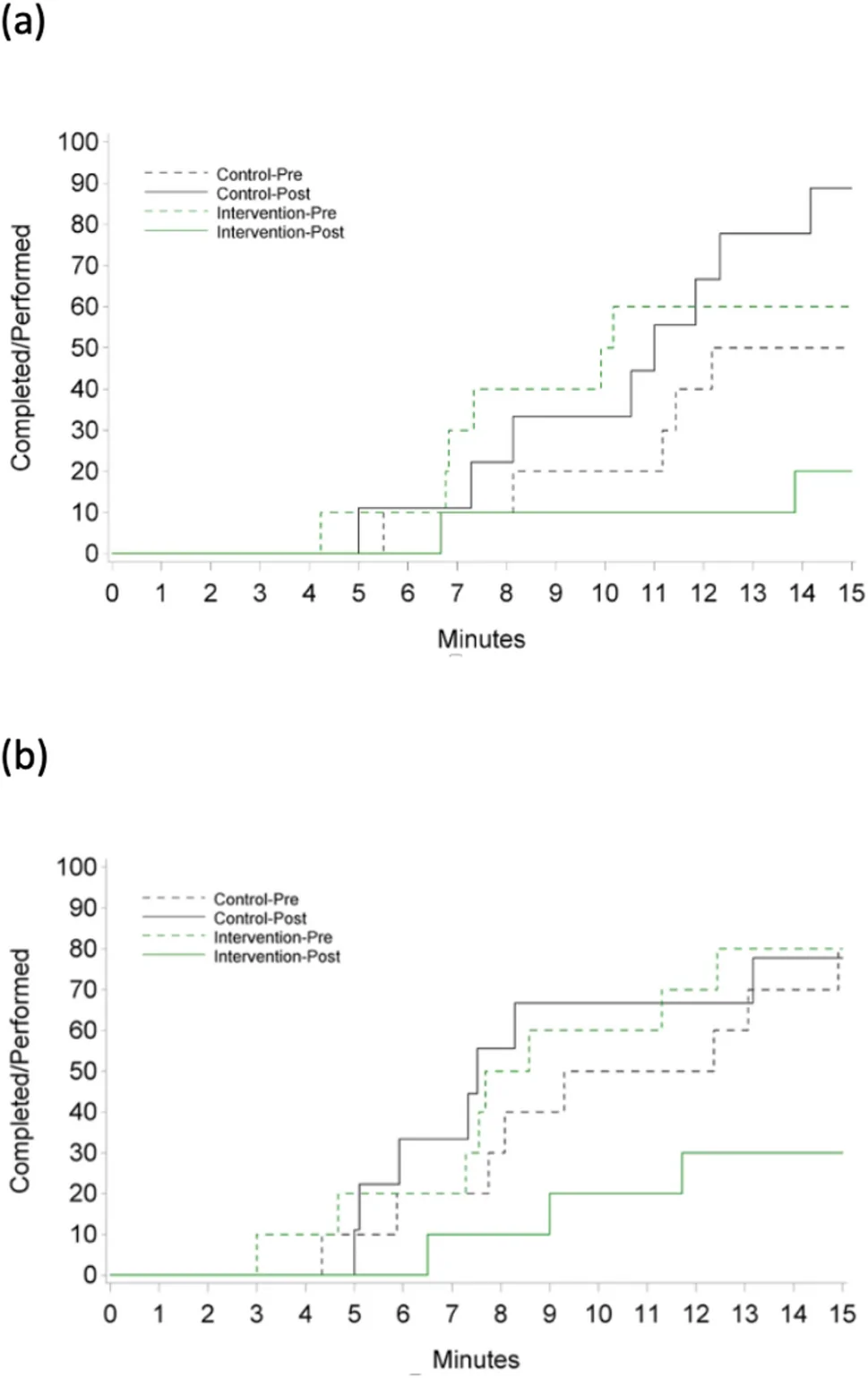
Kaplan–Meier curves illustrating the timing of two critical resuscitation actions. Estimates depicting the cumulative distribution of the time until completion of a specific task: **a** Scenario acute coronary syndrome: timing of mentioning necessity PCI. **b** Scenario pulmonary embolism: timing of mentioning necessity thrombolysis

In the pulmonary embolism scenario, the item ‘Timing of suggestion of considering the need for thrombolysis’ demonstrated a significant pre-post change difference in favor of the control group (Hazard Ratio 0.14, CI: 0.03 to 0.69, p = 0.02, Fig. 3).

Similarly, in the acute coronary syndrome scenario, a significant pre-post change difference was observed favoring the control group concerning the timing of mentioning the necessity of Percutaneous Coronary Intervention (PCI) (Hazard Ratio 0.07, CI: 0.01 to 0.73, *p* = 0.03, Fig. 3).

No other statistically significant between-group pre-post change differences were observed across scenario-specific items.

**Table 3:** Results of critical resuscitation actions analysis

ADDITIONAL ITEMS	CONTROL			INTERVENTION			COMPARISON	
	Est. (95%CI) PRE-phase	Est. (95%CI) POST-phase	Change (95%CI)	Est. (95%CI) PRE-phase	Est. (95%CI) POST-phase	Change (95%CI)	Difference in change (95%CI)	*p*-value
**SCENARIO-INDEPENDENT ITEMS**								
Was a most likely reversible cause identified? *	0.87 (0.57;1.17)	0.92 (0.61;1.23)	0.05 (-0.37;0.47)	0.95 (0.63;1.27)	1.00 (0.68;1.33)	0.06 (-0.38;0.49)	0.01 (-0.60;0.61)	0.99
Was the identified reversible cause correct? *	0.87 (0.57;1.16)	0.89 (0.58;1.20)	0.02 (-0.40;0.44)	0.94 (0.62;1.26)	0.99 (0.67;1.32)	0.05 (-0.38;0.49)	0.03 (-0.58;0.64)	0.92
Did the candidate use the 4 H/4Ts method? *	0.81 (0.53;1.09)	0.80 (0.51;1.08)	-0.01 (-0.41;0.38)	0.60 (0.31;0.88)	0.69 (0.40;0.97)	0.09 (-0.28;0.46)	0.10 (-0.44;0.64)	0.71
Did the candidate consider out loud all the aspects of the 4 H/4T method? *	0.30 (0.14;0.46)	0.38 (0.19;0.57)	0.08 (-0.16;0.31)	0.43 (0.22;0.63)	0.12 (-0.08;0.32)	-0.30 (-0.63;0.02)	-0.38 (-0.78;0.02)	0.06
Was capnography used during resuscitation? *	0.80 (0.52;1.08)	0.83 (0.52;1.13)	0.03 (-0.38;0.43)	0.74 (0.46;1.02)	0.97 (0.65;1.29)	0.23 (-0.18;0.64)	0.20 (-0.38;0.78)	0.49
Timing consideration of reversible causes $	2.7 (1.3;4.0)	3.1 (2.0;4.5)	0.9 (0.6;1.5)	3.7 (2.0;4.6)	2.7 (1.5;3.7)	1.4 (1.0;2.1)	1.6 (0.8;2.9)	0.16
Timing application of capnography. $	6.0 (4.7;7.6)	5.6 (4.4;6.5)	1.1 (0.8;1.6)	5.5 (4.3;6.5)	4.0 (3.2;4.4)	2.2 (1.5;3.0)	1.9 (1.2;3.2)	0.01
Timing first rhythm analysis $	1.0 (0.8;1.2)	0.9 (0.8;1.0)	1.2 (0.8;1.8)	1.0 (0.8;1.2)	1.0 (0.8;1.0)	1.3 (0.9;2.0)	1.1 (0.6;2.1)	0.75
Timing first defibrillation $	1.5 (1.3;1.9)	1.4 (1.2;1.7)	1.5 (1.0;2.3)	1.5 (1.3;1.8)	1.3 (1.1;1.4)	1.5 (1.0;2.5)	1.0 (0.5;1.9)	0.95
**SCENARIO-SPECIFIC ITEMS**								
Scenario Hyperkalemia								
Timing identification reversible cause $	7.1 (5.5;8.4)	8.6 (2.7;10.8)	0.9 (0.3;2.6)	6.0 (3.1;10.8)	10.8 (5.4;11.2)	0.7 (0.3;1.8)	0.8 (0.2;3.1)	0.74
Timing treatment of reversible cause $	8.5 (6.6; ND)	8.4 (4.2;14.9)	1.3 (0.5;3.7)	ND (4.8; ND)	11.5 (5.4;12.6)	1.4 (0.5;4.3)	1.1 (0.2;5.0)	0.92
Scenario Pulmonary Embolism								
Timing identification reversible cause $	7.5 (2.6;9.0)	6.7 (3.4;9.9)	1.1 (0.5;2.4)	6.4 (1.5;7.4)	6.4 (3.7;8.4)	1.0 (0.4;2.7)	1.0 (0.3;3.4)	0.93
Timing treatment of reversible cause $	ND (8.7; ND)	ND (5.3; ND)	2.0 (0.6;6.7)	ND (5.4; ND)	ND (8.6; ND)	0.3 (0.0;3.4)	0.2 (0.0;2.3)	0.17
Timing of suggestion of considering the need for trombolysis $	10.8 (4.3;14.9)	7.5 (5.0; ND)	1.7 (0.6;4.7)	8.1 (3.0;12.4)	ND (6.5; ND)	0.2 (0.1;0.8)	0.1 (0.0;0.7)	0.02
Scenario Acute Coronary Syndrome								
Timing identification reversible cause $	9.3 (4.5; ND)	6.4 (3.9;8.5)	2.6 (0.8;8.5)	7.23 (2.9;9.6)	6.0 (4.3;10.2)	1.0 (0.4;2.5)	0.4 (0.1;1.8)	0.22
Timing treatment of reversible cause $	ND (5.0; ND)	ND (7.2; ND)	1.9 (0.3;10.5)	ND (4.3; ND)	ND (5.9; ND)	1.3 (0.3;6.1)	0.7 (0.1;6.9)	0.78
Timing of mentioning the necessity of PCI $	ND (5.5; ND)	11.0 (5.0;14.2)	2.4 (0.8;7.0)	10.0 (4.2; ND)	ND (6.7; ND)	0.2 (0.0;1.1)	0.1 (0.0;0.7)	0.03
Scenario TCA intoxication								
Timing identification reversible cause $	7.1 (3.8;8.6)	10.4 (6.2;11.2)	1.0 (0.4;2.8)	11.2 (2.8;11.6)	9.0 (3.4;11.1)	1.1 (0.5;2.6)	1.1 (0.3;4.5)	0.85
Timing treatment of reversible cause $	12.6 (5.5; ND)	11.93 (8.18; ND)	1.4 (0.4;4.9)	12.7 (7.8; ND)	12.3 (3.8; ND)	1.0 (0.4;3.0)	0.7 (0.1;3.7)	0.69

*Est.* estimate, *ND* not determined

(*) estimates (95%CI) are proportions (or changes and differences in changes of proportions) obtained from an absolute risk model

($) estimates (95%CI) are median minutes (95%CI). The changes and differences in changes refer to hazard ratios obtained from Cox regressions. A hazard ratio for the change higher than 1 refers to a shorter time

### Descriptive information ECPR protocol [Additional file 8]

The mean score on the ECPR protocol knowledge test was 94,5%. All participants in the intervention group successfully met the 80% threshold required to advance to the post-phase scenarios.

In scenarios requiring ECPR protocol activation, it was declared in 39 of 40 cases (97.5%). Among these, three participants violated the protocol: two involved delayed declaration, and one failed to prepare the patient for prompt transport after declaring the protocol. The median time for the ECPR protocol declaration was 7.5 min.

## Discussion

To the best of our knowledge, this is the first randomized controlled simulation study to evaluate the impact of introducing a prehospital ECPR protocol on resuscitation quality and team leader performance during advanced life support. The primary outcome (modified Peltonen score) showed no significant difference in overall team leader performance between groups. Yet, secondary analyses showed delays in addressing reversible causes of cardiac arrest in the intervention group.

These observations suggest a cognitive shift in the intervention group, away from diagnostic reasoning toward a more protocol-driven mode of management reasoning resulting in action. Participants appeared to prioritize immediate execution of the ECPR protocol, focusing on the procedural steps required for activation, rather than on identifying and treating reversible causes of cardiac arrest. While this management and action-oriented mindset facilitates timely protocol execution, it may come at the cost of reduced diagnostic attention and delayed therapeutic decisions. Such delays could negatively impact patient outcomes, and there is a potential risk of initiating ECPR in cases where less invasive interventions might have been sufficient to achieve return of spontaneous circulation. Although this study was conducted in a physician-led EMS system, the observed delays may also potentially occur in paramedic-based systems where strict protocol adherence could similarly compete with diagnostic reasoning.

From a theoretical standpoint, the observed findings can be interpreted using the concept of a management script, introduced by Parsons et al. to describe the mental schema that clinicians develop and use during medical decision-making [19]. Within the context of this study, the management script for the diagnosis of “cardiac arrest” can be understood as comprising several layers including execution of the ECPR protocol, continuation of high-quality ALS, and treatment of any identified reversible causes of arrest. At the same time, refinement of diagnostic reasoning should continue through the search for such reversible causes. Our findings suggest that participants performing ECPR tended to prioritize the layer related to ECPR protocol execution. This focus appears to consume a substantial portion of the available cognitive bandwidth, leaving less capacity for other essential layers of the management script and for the concurrent process of diagnostic reasoning.

These findings underscore the need for education that integrates diagnostic and management reasoning with timely ECPR activation, ensuring reversible causes of cardiac arrest are identified and addressed without delaying treatment.

Simulation-based education and training, widely recognized as an effective tool for developing and enhancing skills in acute care medicine, can play a pivotal role in achieving these goals [13, 14, 20–25]. By replicating high-pressure scenarios, simulation allows health care professionals to practice balancing timely protocol execution with critical diagnostic reasoning, ensuring they are well-prepared to manage the complexities of ECPR protocol implementation while minimizing potential risks.

In addition, cognitive aids may help team leaders maintain cognitive focus on identifying and addressing reversible causes of cardiac arrest during ECPR implementation. These tools can help ensure that critical steps in diagnosis and treatment are not overlooked, even in high-stress, action-focused situations [26–29].

Finally, Crew Resource Management (CRM) training may help mitigate cognitive overload by fostering non-technical skills such as task prioritization, shared mental models, and situational awareness [30].

As medicine continues to advance, the expanding range of diagnostic and therapeutic options inevitably increases the complexity of acute care. This growing complexity may translate into greater cognitive load for clinicians, particularly in time-critical situations, thereby increasing the risk of errors or suboptimal performance [31]. Investigating the impact of expanded protocols and additional decision layers on clinical reasoning and patient outcomes in real-world settings is methodologically and ethically challenging. For this reason, in the present study simulation was used as a methodological platform to examine the performance implications of introducing additional protocol complexity under controlled conditions.

Simulation is particularly well suited to address such research questions because it eliminates the risk of patient harm while allowing deliberate manipulation of clinical complexity. Also, unlike real-world environments, simulation allows controlled modification of a single variable within standardized conditions, thereby enabling more precise examination of its relationship with measured outcomes.

In the present study, both scenarios and team member behaviour were deliberately standardized to enable focused assessment of how introduction of the ECPR protocol influenced team leader performance, while minimizing confounding variability. Such experimental control, facilitating a more precise assessment of the variable of interest, would not be feasible in real-world prehospital practice, where team dynamics, clinical presentations, and environmental conditions cannot be standardized. However, this methodological choice involves inherent trade-offs. By standardizing team member behaviour to isolate leadership performance, natural team dynamics were inevitably reduced. In real teams, members may anticipate tasks, contribute independent observations, and compensate for cognitive overload in the leader. This limitation may restrict the generalizability of the herein observed team leader performance to real-world resuscitation teams.

Future research should further examine how advanced resuscitation protocols influence team performance within intact multidisciplinary teams, taking natural team dynamics into account. Incorporating direct measures of cognitive load and evaluating longer-term retention of ECPR training may provide additional insight into how protocol complexity affects decision-making and performance over time. In addition, future research should specifically examine whether targeted simulation training, CRM-based interventions, and cognitive aids can mitigate potential performance decrements associated with increased protocol complexity.

### Limitations

Each simulation was rated by one of four assessors, which may have introduced rater bias due to subjective scoring differences. To minimize this risk, all raters received joint training, used a standardized scoring tool, and held regular consensus meetings to ensure consistent scoring.

We did not stratify randomization by discipline, leading to an imbalance in the control and intervention groups’ medical specialties; this imbalance was addressed in the statistical models. Additionally, the study groups were not stratified by training level (junior vs. senior doctors), leading to a slight imbalance between groups in terms of clinical experience. While these imbalances may have introduced noise, previous ALS performance studies have shown that clinical discipline and years of experience are not strong independent predictors of simulated resuscitation quality compared with structured algorithm training [32–34]. Therefore, these imbalances are unlikely to have systematically biased the comparison between groups. Moreover, “senior” status was defined as > 3 years of clinical practice and did not necessarily correspond to independent attending status, as some participants were still in specialist training.

Prehospital clinical exposure during residency differs between specialties in Belgium: it is for example routinely included in Anesthesia and Emergency Medicine training, but not systematically implemented in Internal Medicine training. Variability in prior prehospital experience may therefore have influenced performance in the simulated setting.

Furthermore, the Peltonen instrument was adapted to a team-leader-only setting. While this modification was theoretically grounded and focused on leadership-dependent items, the psychometric properties of this reduced version have not been formally validated. Therefore, the findings based on this adapted instrument should be interpreted as exploratory.

Another limitation of this study is the deliberate standardization of team member behaviour to enable focused assessment of team leader performance within a simulated setting. Team members were represented by medical students who performed standardized roles. Although this approach enhanced experimental control, it inherently reduced natural team dynamics. This limits the generalizability of our findings to fully intact clinical teams. Nonetheless, in the Belgian prehospital system, cardiac arrest teams typically consist of one nurse-paramedic, two ambulance operators, and a single physician who assumes clear leadership. Given this structure, we believe that focusing on the physicians’ performance provides a contextually meaningful measure of resuscitation quality in this simulated setting, though future studies should also evaluate full-team interactions in actual practice.

Additionally, as with all simulation-based research, external validity is limited. Performance in a simulated environment may differ from real-world prehospital resuscitation, and findings should therefore be interpreted with caution when extrapolating to clinical practice.

Furthermore, the ECPR training was delivered within a relatively short timeframe before the post-intervention scenarios, which may limit generalizability. In real-world practice, longer intervals between training and clinical application could alter cognitive demands and performance.

Finally, this study was not powered to test for non-inferiority. Although no statistically significant difference was observed in the primary outcome, this does not confirm equivalence. The findings should be interpreted as exploratory.

## Conclusions

In this simulation study, combining a prehospital ECPR protocol with ALS resulted in overall performance comparable to standard ALS alone. However, our findings suggest a shift in team leader behaviour toward a more protocol-driven mode of ECPR actions. The ECPR protocol appeared to dominate cognitive resources, potentially displacing timely recognition and treatment of reversible causes of cardiac arrest. These findings underline the importance of embedding future ECPR education within a broader cognitive framework that reinforces diagnostic and therapeutic vigilance alongside procedural execution.

## Supplementary Information

Additional file 1. Scenario scripts.

Additional file 2. Flowchart participants.

Additional file 3. Inclusion and Exclusion Criteria ECPR protocol.

Additional file 4. ECPR knowledge test.

Additional file 5. Peltonen Score modification.

Additional file 6. Extended results of critical resuscitation actions.

Additional file 7. Excluded data.

Additional file 8. Descriptive information ECPR protocol execution.

## Data Availability

Data supporting the findings of this study are available from the corresponding author upon reasonable request.

## Abbreviations

ALS: Advanced Life Support
ECPR: Extracorporeal Cardiopulmonary Resuscitation
ECMO: Extracorporeal Membrane Oxygenation
ERC: European Resuscitation Council
