# Supplementary material for: Evaluating the impact of implementing an ECPR protocol on prehospital resuscitation quality: a randomized controlled simulation study
Source: Adv Simul (Lond). 2026 Jul 9;11:69. doi: 10.1186/s41077-026-00458-3 (PMC13637304; doi:10.1186/s41077-026-00458-3)
Supplement: Supplementary file 1 [file 41077_2026_458_MOESM2_ESM.pdf]

Appendix 2: Flowchart participants

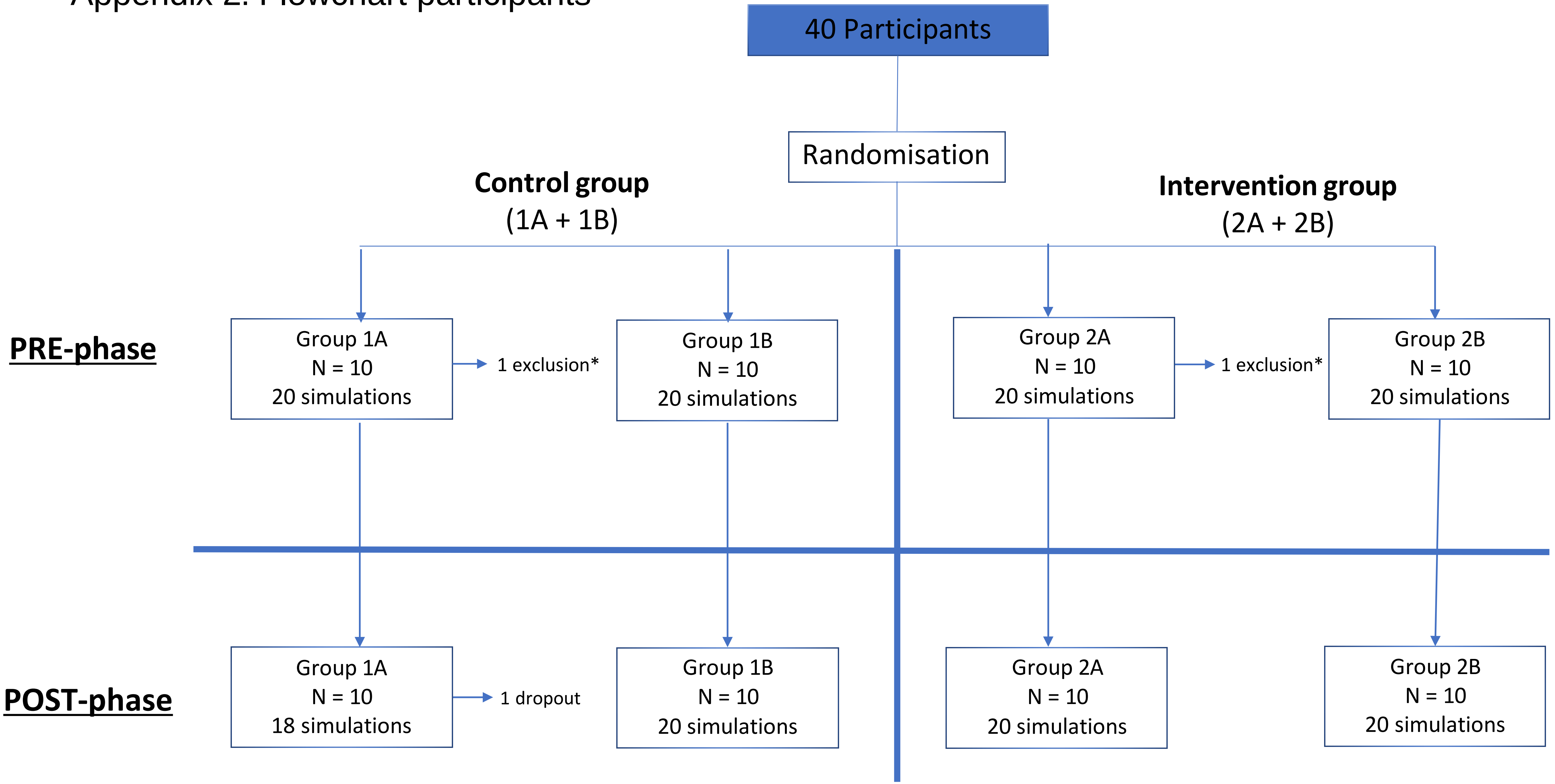

* Excluded participants: 2 participants were excluded from the study due to technical failure during the pre-phase. These participants were replaced by two new participants.
