# Supplementary material for: Evaluating the impact of implementing an ECPR protocol on prehospital resuscitation quality: a randomized controlled simulation study
Source: Adv Simul (Lond). 2026 Jul 9;11:69. doi: 10.1186/s41077-026-00458-3 (PMC13637304; doi:10.1186/s41077-026-00458-3)
Supplement: Supplementary file 3 [file 41077_2026_458_MOESM3_ESM.docx]

**Additional File 3: Inclusion and Exclusion Criteria for ECPR in the introduced protocol**

## Inclusion Criteria

- Age <65–70 years (biological age considered over chronological age)
- Witnessed cardiac arrest
- Initiation of chest compressions within 5 minutes of arrest
- Estimated time between cardiac arrest and ECMO initiation <60 minutes
- Initial rhythm was not asystole

## Exclusion Criteria

- Known pre-existing do-not-intubate (DNI) or do-not-resuscitate (DNR) order
- Significant pre-existing comorbidities
- Multi-organ failure
- Vasopressor-refractory shock
- Low-flow time >60 minutes
- Exceptionally extendable up to 90 minutes in cases of:
- Signs of life during resuscitation
- Hypothermia
- Intoxication
- High end-tidal CO₂ (EtCO₂) levels

Note: also think about relative contra-indications (Moderate AI (2-3/4),..)
