# Supplementary material for: Evaluating the impact of implementing an ECPR protocol on prehospital resuscitation quality: a randomized controlled simulation study
Source: Adv Simul (Lond). 2026 Jul 9;11:69. doi: 10.1186/s41077-026-00458-3 (PMC13637304; doi:10.1186/s41077-026-00458-3)
Supplement: Supplementary file 4 [file 41077_2026_458_MOESM4_ESM.docx]

**Additional File 4: ECPR knowledge test: Dutch/English**

**Dutch version**

1. De definitieve beslissing tot ECPR dient genomen te worden binnen de 25 minuten na aankomst MUG.

**Fout.**

2. Een afgetekende DNR betekent een exclusie voor ECPR.

**Juist.**

3. Thoraxcompressies moeten ten laatste binnen de 5 minuten na collaps gestart zijn om in aanmerking te komen voor ECPR.

**Juist.**

4. Wanneer je het ECPR protocol activeert geef je niet meer dan 5 mg adrenaline in totaal.

**Juist.**

5. Het ECPR protocol wordt pre-hospitaal geactiveerd bij een patiënt in cardiaal arrest met een schokbaar ritme. Over 40 minuten kan deze patiënt op ECMO staan. Je blijft proberen deze patiënt te defibrilleren om de twee minuten tot hij effectief op ECMO staat.
   **Fout.**
6. Een aorta-insufficiëntie is een relatieve contra-indicatie voor ECPR.

**Juist.**

7. Een cardiaal arrest op basis van een intoxicatie is op zich een goede indicatie voor ECPR.
   **Juist.**
8. Een man van 55 jaar met een intra-abdominale bloeding gaat in hypovoleme shock en vervolgens in cardiaal arrest op basis van hypovolemie. Op basis van deze ethiologie is dit een goede kandidaat voor ECPR.
   **Fout.**
9. Wanneer ECPR wordt uitgevoerd dient de patiënt ten laatste laatste twee uur na collaps op ECMO te staan.

**Fout.**

10. Capnografie is een belangrijke parameter tijdens de reanimatie, ook na afkondiging van het ECPR protocol.
    **Juist.**

**English version**

1. The definitive decision for ECPR must be made within 25 minutes after the arrival of the MUG (= Belgian prehospital team consisting of a Medical Doctor and a nurse). **False.**
2. A signed DNR (Do Not Resuscitate) order implies exclusion for ECPR.
   **True.**
3. Chest compressions should be initiated no later than 5 minutes after collapse to be eligible for ECPR.
   **True.**
4. When activating the ECPR protocol, you do not administer more than a total of 5 mg of adrenaline.
   **True.**
5. The ECPR protocol is activated in the pre-hospital setting for a patient in cardiac arrest with a shockable rhythm. Within 40 minutes, this patient can be on ECMO.
   You should continue attempting to defibrillate this patient every two minutes until they are truly on ECMO.
   **False.**
6. Aortic insufficiency is a relative contraindication for ECPR.
   **True.**
7. Cardiac arrest due to intoxication is a good indication for ECPR.
   **True.**
8. A 55-year-old male with intra-abdominal bleeding suffers hypovolemic shock and subsequently has a cardiac arrest due to hypovolemia. Based on this etiology of cardiac arrest, this is a good candidate for ECPR.
   **False.**
9. When ECPR is performed, the patient should be on ECMO no later than two hours after cardiac arrest.
   **False.**
10. Capnography is an important parameter during resuscitation, even after the activation of the ECPR perotocol.
    **True.**
