# Supplementary material for: Evaluating the impact of implementing an ECPR protocol on prehospital resuscitation quality: a randomized controlled simulation study
Source: Adv Simul (Lond). 2026 Jul 9;11:69. doi: 10.1186/s41077-026-00458-3 (PMC13637304; doi:10.1186/s41077-026-00458-3)
Supplement: Supplementary file 5 [file 41077_2026_458_MOESM5_ESM.docx]

**Additional File 5: Peltonen Score modification**

Items in red: not evaluated in this study.

| 1 | Was the need for cardiopulmonary resuscitation immediately recognised? |
| --- | --- |
| 2 | Was help called for immediately? |
| 3 | Were chest compressions initiated immediately? |
| 4 | Was the airway opened and breathing assessed? |
| 5 | Was the patient lifted on a firm surface or was a backboard placed when necessary? |
| 6 | Was the compression technique correct? |
| 7 | Was the compression rate 100–120 per min.? |
| 8 | Was the compression-ventilation rate correct? |
| 9 | Was the compression depth correct? |
| 10 | Was there complete chest recoil between compressions? |
| 11 | Was the number of interruptions in the chest compressions minimised? |
| 12 | Was the length of the interruptions in the chest compressions < 10 s.? |
| 13 | Was the CPR quality evaluated? |
| 14 | Was the compressor rotation adequate? |
| 15 | Was 100% oxygen administered as soon as possible? |
| 16 | Was the airway ensured using appropriate equipment as soon as possible? |
| 17 | Was placement of an advanced airway ensured? |
| 18 | Did ventilation actualise according to guidelines? |
| 19 | Were the pads placed as soon as the defibrillator was on site? |
| 20 | Were the pads placed correctly on the patient’s chest? |
| 21 | Was the rhythm (VT/VF/ASY/PEA) correctly identified? |
| 22 | Did defibrillation actualise according to guidelines if the rhythm was VF/VT |
|  | OR were compressions immediately continued if the rhythm was PEA/ASY? |
| 23 | Was rhythm analysis performed with 2 min. intervals? |
| 24 | Was the resuscitation algorithm followed? |
| 25 | Was a parenteral medication route inserted as soon as possible? |
| 26 | Was medication use appropriate? |
| 27 | Was a suitable amount of fluid given after medication to facilitate the drug flow? |
| 28 | Were medicines administered as ordered? |
| 29 | Were possible causes for the cardiac arrest analysed? |
| 30 | Were reversible causes for the cardiac arrest treated? |
| 31 | Were tasks prioritised appropriately? |
| 32 | Was vital organ support optimised if ROSC was attained? |
| 33 | Was an appropriate care plan made for the patient if ROSC was attained? |
| 34 | Were there a suitable number of professionals present? |
| 35 | Was it clear who led the team? |
| 36 | Were all team members’ roles clear? |
| 37 | Were all roles appropriate for the team members? |
| 38 | Did the leader continuously outline a plan of action? |
| 39 | Did a team member continuously maintain the overview of the situation? |
| 40 | Did a team member continuously monitor the patient’s condition? |
| 41 | Did a team member continuously give corrective feedback about the quality of CPR? |
| 42 | Did a team member continuously follow -up the adherence to the resuscitation algorithm? |
| 43 | Did a team member continuously inform the others when it was time for an |
|  | intervention according to the resuscitation algorithm? |
| 44 | Did a team member continuously inform others about changes in the patient’s condition? |
| 45 | Did the team members carry out their responsibilities? |
| 46 | Did the team members ask for help when needed? |
| 47 | Did the team members assist each other when needed? |
| 48 | Was the situation handled calmly? |
| 49 | Did the team members behave professionally towards each other? |
| 50 | Did the team members behave professionally towards the patient? |
| 51 | Did the team members correct each other when necessary? |
| 52 | Did the team members inform others about their observations and insights? |
| 53 | Was the leader open to suggestions from other team members? |
| 54 | Was communication between the team members clear? |
| 55 | Was ‘closed loop communication’ used? |
| 56 | Was the number of chest compressions counted aloud? |
| 57 | Was relevant patient information shared effectively between professionals? |
| 58 | Was additional patient information made available? |
| 59 | Was the cardiac resuscitation process documented? |
| 60 | Was patient integrity protected? |
| 61 | Was patient information handled discretely? |
| 62 | Were laymen guided away from the resuscitation site when necessary? |
| 63 | Were laymen given support when necessary? |
| 64 | Did the team members protect themselves adequately? |
| 65 | Did the team find the site of the cardiac resuscitation quickly and easily? |
| 66 | Did the team members have the right equipment? |
| 67 | Did the team members know how to use the equipment? |
| 68 | Were the team members able to take into consideration the physical resources and restraints of the site? |
| 69 | Were the team members working ergonomically? |
