# Supplementary material for: Evaluating the impact of implementing an ECPR protocol on prehospital resuscitation quality: a randomized controlled simulation study
Source: Adv Simul (Lond). 2026 Jul 9;11:69. doi: 10.1186/s41077-026-00458-3 (PMC13637304; doi:10.1186/s41077-026-00458-3)
Supplement: Supplementary file 6 [file 41077_2026_458_MOESM6_ESM.docx]

**Additional File 6: Extended Results critical resuscitation actions analysis**

| **ADDITIONAL ITEMS** | **CONTROL** | | | **INTERVENTION** | | | **COMPARISON** | |
| --- | --- | --- | --- | --- | --- | --- | --- | --- |
|  | **Est. (95%CI) PRE-phase** | **Est. (95%CI) POST-phase** | **Change (95%CI)** | **Est. (95%CI) PRE-phase** | **Est. (95%CI) POST-phase** | **Change (95%CI)** | **Difference in change (95%CI)** | **p-value** |
| **Scenario-independent items** |  |  |  |  |  |  |  |  |
| Was a most likely reversible cause identified?* | 0.87 (0.57;1.17) | 0.92 (0.61;1.23) | 0.05 (-0.37;0.47) | 0.95 (0.63;1.27) | 1.00 (0.68;1.33) | 0.06 (-0.38;0.49) | 0.01 (-0.60;0.61) | 0.99 |
| Was the identified reversible cause correct? * | 0.87 (0.57;1.16) | 0.89 (0.58;1.20) | 0.02 (-0.40;0.44) | 0.94 (0.62;1.26) | 0.99 (0.67;1.32) | 0.05 (-0.38;0.49) | 0.03 (-0.58;0.64) | 0.92 |
| Did the candidate use the 4H/4Ts method? * | 0.81 (0.53;1.09) | 0.80 (0.51;1.08) | -0.01 (-0.41;0.38) | 0.60 (0.31;0.88) | 0.69 (0.40;0.97) | 0.09 (-0.28;0.46) | 0.10 (-0.44;0.64) | 0.71 |
| Did the candidate consider out loud all the aspects of the 4H/4T method?* | 0.30 (0.14;0.46) | 0.38 (0.19;0.57) | 0.08 (-0.16;0.31) | 0.43 (0.22;0.63) | 0.12 (-0.08;0.32) | -0.30 (-0.63;0.02) | -0.38 (-0.78;0.02) | 0.06 |
| Was capnography used during resuscitation?* | 0.80 (0.52;1.08) | 0.83 (0.52;1.13) | 0.03 (-0.38;0.43) | 0.74 (0.46;1.02) | 0.97 (0.65;1.29) | 0.23 (-0.18;0.64) | 0.20 (-0.38;0.78) | 0.49 |
| Timing consideration of reversible causes $ | 2.73 (1.33;3.97) | 3.14 (2.03;4.50) | 0.91 (0.57;1.46) | 3.71 (2.02;4.58) | 2.68 (1.52;3.67) | 1.41 (0.96;2.06) | 1.55 (0.84;2.87) | 0.16 |
| Timing application of capnography. $ | 5.98 (4.73;7.58) | 5.62 (4.38;6.50) | 1.13 (0.78;1.64) | 5.53 (4.33;6.47) | 4.01 (3.17;4.42) | 2.16 (1.54;3.02) | 1.90 (1.15;3.16) | 0.01 |
| Timing first rhythm analysis $ | 0.99 (0.83;1.17) | 0.91 (0.75;1.02) | 1.17 (0.76;1.81) | 1.00 (0.83;1.17) | 0.95 (0.75;1.03) | 1.30 (0.85;2.00) | 1.11 (0.58;2.12) | 0.75 |
| Timing first defibrillation $ | 1.53 (1.32;1.87) | 1.40 (1.17;1.67) | 1.50 (0.96;2.32) | 1.48 (1.27;1.83) | 1.32 (1.13;1.42) | 1.53 (0.95;2.46) | 1.02 (0.54;1.94) | 0.95 |
|  |  |  |  |  |  |  |  |  |
| **Scenario-specific items** |  |  |  |  |  |  |  |  |
| **Scenario Hyperkalemia** |  |  |  |  |  |  |  |  |
| Was a most likely reversible cause identified?* | 0.80 (0.22;1.38) | 0.80 (0.21;1.38) | -0.00 (-0.82;0.81) | 0.79 (0.14;1.45) | 1.00 (0.35;1.65) | 0.21 (-0.69;1.10) | 0.21 (-1.01;1.43) | 0.73 |
| Was the identified reversible cause correct?* | 0.80 (0.22;1.38) | 0.80 (0.21;1.38) | -0.00 (-0.82;0.81) | 0.79 (0.14;1.45) | 1.00 (0.35;1.65) | 0.21 (-0.69;1.10) | 0.21 (-1.01;1.43) | 0.73 |
| Correct treatment of correct identified reversible cause?* | 0.58 (0.09;1.07) | 0.63 (0.11;1.15) | 0.05 (-0.67;0.78) | 0.53 (0.01;1.06) | 0.71 (0.16;1.26) | 0.17 (-0.53;0.88) | 0.12 (-0.92;1.16) | 0.81 |
| Timing identification reversible cause $ | 7.08 (5.52;8.42) | 8.63 (2.70;10.75) | 0.92 (0.33;2.60) | 6.04 (3.05;10.75) | 10.78 (5.42;11.17) | 0.73 (0.30;1.83) | 0.80 (0.21;3.09) | 0.74 |
| Timing treatment of reversible cause $ | 8.46 (6.55; ND) | 8.42 (4.17;14.92) | 1.29 (0.46;3.67) | ND (4.77; ND) | 11.53 (5.42;12.57) | 1.40 (0.46;4.29) | 1.09 (0.24;5.01) | 0.92 |
|  |  |  |  |  |  |  |  |  |
| **Scenario Pulmonary Embolism** |  |  |  |  |  |  |  |  |
| Was a most likely reversible cause identified?* | 1.00 (0.34;1.66) | 1.00 (0.31;1.69) | 0.00 (-0.94;0.94) | 1.00 (0.34;1.66) | 1.00 (0.27;1.73) | 0.00 (-0.94;0.94) | 0.00 (-1.33;1.33) | 1.00 |
| Was the identified reversible cause correct?* | 0.98 (0.34;1.63) | 0.86 (0.20;1.51) | -0.13 (-1.03;0.78) | 0.98 (0.34;1.63) | 0.94 (0.22;1.66) | -0.04 (-0.99;0.91) | 0.08 (-1.22;1.39) | 0.90 |
| Did teamleader make suggestion of considering trombolysis asap?* | 0.87 (0.28;1.46) | 0.81 (0.20;1.43) | -0.06 (-0.88;0.77) | 0.78 (0.22;1.36) | 0.35 (-0.10;0.79) | -0.44 (-1.13;0.25) | -0.39 (-1.45;0.68) | 0.47 |
| Timing identification reversible cause $ | 7.47 (2.62;9.02) | 6.70 (3.42;9.93) | 1.07 (0.49;2.35) | 6.44 (1.50;7.43) | 6.40 (3.72;8.42) | 1.01 (0.39;2.66) | 0.95 (0.26;3.38) | 0.93 |
| Timing treatment of reversible cause $ | ND (8.70; ND) | ND (5.33; ND) | 1.95 (0.56;6.73) | ND (5.38; ND) | ND (8.62; ND) | 0.29 (0.03;3.36) | 0.15 (0.01;2.26) | 0.17 |
| Timing of suggestion of considering the need for trombolysis $ | 10.83 (4.33;14.92) | 7.52 (5.00; ND) | 1.65 (0.58;4.74) | 8.13 (3.00;12.43) | ND (6.50; ND) | 0.23 (0.06;0.81) | 0.14 (0.03;0.69) | 0.02 |
|  |  |  |  |  |  |  |  |  |
| **Scenario Acute Coronary Syndrome** |  |  |  |  |  |  |  |  |
| Was a most likely reversible cause identified?* | 0.65 (0.08;1.22) | 0.89 (0.25;1.53) | 0.24 (-0.62;1.10) | 1.00 (0.35;1.65) | 0.96 (0.25;1.66) | -0.04 (-0.98;0.89) | -0.28 (-1.58;1.02) | 0.66 |
| Was the identified reversible cause correct?* | 0.65 (0.08;1.22) | 0.89 (0.25;1.53) | 0.24 (-0.62;1.10) | 1.00 (0.35;1.65) | 0.96 (0.25;1.66) | -0.04 (-0.98;0.89) | -0.28 (-1.58;1.02) | 0.66 |
| Correct treatment of correct identified reversible cause?* | -0.07 (-0.60;0.47) | 0.30 (-0.20;0.79) | 0.36 (-0.38;1.11) | 0.41 (-0.07;0.90) | 0.33 (-0.18;0.84) | -0.09 (-0.93;0.75) | -0.45 (-1.66;0.76) | 0.45 |
| Did teamleader mention neccesity to perform PCI asap?* | 0.51 (0.03;0.10) | 0.89 (0.25;1.53) | 0.38 (-0.41;1.17) | 0.60 (0.09;1.11) | 0.20 (-0.18;0.57) | -0.40 (-1.00;0.19) | -0.79 (-1.78;0.20) | 0.12 |
| Timing identification reversible cause $ | 9.29 (4.50; ND) | 6.35 (3.87;8.48) | 2.60 (0.80;8.48) | 7.23 (2.87;9.63) | 6.00 (4.25;10.17) | 1.01 (0.41;2.49) | 0.39 (0.09;1.77) | 0.22 |
| Timing treatment of reversible cause $ | ND (5.00; ND) | ND (7.17; ND) | 1.86 (0.33;10.45) | ND (4.33; ND) | ND (5.92; ND) | 1.34 (0.30;6.07) | 0.72 (0.08;6.86) | 0.78 |
| Timing of mentioning the necessity of PCI $ | ND (5.50; ND) | 11.00 (5.00;14.17) | 2.40 (0.83;6.98) | 10.04 (4.23; ND) | ND (6.77; ND) | 0.18 (0.03;1.14) | 0.07 (0.01;0.73) | 0.03 |
|  |  |  |  |  |  |  |  |  |
| **Scenario TCA intoxication** |  |  |  |  |  |  |  |  |
| Was a most likely reversible cause identified?* | 1.00 (0.36;1.64) | 1.00 (0.34;1.66) | 0.00 (-0.91;0.91) | 1.00 (0.28;1.72) | 1.00 (0.35;1.65) | 0.00 (-0.94;0.94) | 0.00 (-1.32;1.32) | 1.00 |
| Was the identified reversible cause correct?* | 1.00 (0.36;1.64) | 1.00 (0.34;1.66) | 0.00 (-0.91;0.91) | 1.00 (0.28;1.72) | 1.00 (0.35;1.65) | 0.00 (-0.94;0.94) | 0.00 (-1.32;1.32) | 1.00 |
| Correct treatment of correct identified reversible cause?* | 0.63 (0.12;1.13) | 0.67 (0.16;1.17) | 0.04 (-0.64;0.72) | 0.60 (0.10;1.10) | 0.45 (0.04;0.85) | -0.16 (-0.70;0.39) | -0.19 (-1.08;0.69) | 0.66 |
| Was NaBic administered?* | 1.38 (0.62;2.15) | 1.25 (0.51;1.98) | -0.14 (-1.20;0.92) | 1.50 (0.60;2.36) | 1.36 (0.60;2.13) | -0.12 (-1.26;1.02) | 0.02 (-1.55;1.60) | 0.98 |
| Timing identification reversible cause $ | 7.08 (3.83;8.55) | 10.35 (6.17;11.23) | 1.00 (0.36;2.80) | 11.18 (2.83;11.63) | 8.96 (3.43;11.07) | 1.14 (0.50;2.57) | 1.14 (0.29;4.46) | 0.85 |
| Timing treatment of reversible cause $ | 12.63 (5.47; ND) | 11.93 (8.18; ND) | 1.44 (0.42;4.88) | 12.70 (7.75; ND) | 12.30 (3.75; ND) | 1.03 (0.35;3.02) | 0.72 (0.14;3.68) | 0.69 |
| Est.: estimate. ND: not determined. (*) estimates (95%CI) are proportions (or changes and differences in changes of proportions) obtained from an absolute risk model.  ($) estimates (95%CI) are median minutes (95%CI). The changes and differences in changes refer to hazard ratios obtained from Cox regressions. A hazard ratio for the change higher than 1 refers to a shorter time. | | | | | | | | |

Estimates (and 95% confidence intervals), pre-post changes, and comparison of the changes obtained from absolute risk models or Cox regressions, depending on the item. The results are presented for the total sample.
