# Supplementary material for: Evaluating the impact of implementing an ECPR protocol on prehospital resuscitation quality: a randomized controlled simulation study
Source: Adv Simul (Lond). 2026 Jul 9;11:69. doi: 10.1186/s41077-026-00458-3 (PMC13637304; doi:10.1186/s41077-026-00458-3)
Supplement: Supplementary file 7 [file 41077_2026_458_MOESM7_ESM.docx]

**Additional File 7: Excluded data**

**Simulation 2 – Participant 28:**

- Was a parenteral medication route inserted as soon as possible? (question Peltonen)

Reason of exclusion:

Error against scenario script: IV access succesful at first attempt (should have been unsuccesful).

**Simulation 4 – Participant 11**

- Timing of the identification of the most likely reversible cause of cardiac arrest
- Timing of treatment of the identified reversible cause of cardiac arrest

Reason of exclusion:

Error against scenario script: At 6’40’’ ambulance coworker already mentions finding empty medications box of TCA (should only be at minute 11).

**Simulation 4 – Participant 33**

- Timing of the identification of the most likely reversible cause of cardiac arrest
- Timing of treatment of the identified reversible cause of cardiac arrest

Reason of exclusion:

Error against scenario script: At arrival of medical team the bystander immediately mentions the patient having pain on the chest pre-collaps. (should not have been said spontaneously).

**Participants 7 and 9:**

- All data

Reason of exclusion:

Technical failure recording the pre-phase.
