# Supplementary material for: Evaluating the impact of implementing an ECPR protocol on prehospital resuscitation quality: a randomized controlled simulation study
Source: Adv Simul (Lond). 2026 Jul 9;11:69. doi: 10.1186/s41077-026-00458-3 (PMC13637304; doi:10.1186/s41077-026-00458-3)
Supplement: Supplementary file 8 [file 41077_2026_458_MOESM8_ESM.docx]

**Appendix 8: Descriptive information ECPR protocol execution (intervention group)**

### ***Was the ECPR protocol activated (defined as a call to floormanager of emergency department to activate the protocol) ?***

|  | | *Control* | *Intervention* |
| --- | --- | --- | --- |
| PRE | n/N (%) | 0/ 0 | 0/ 0 |
| POST | n/N (%) | 0/ 0 | 39/ 40 ( 97.50%) |
|  | | | |

### ***Did the teamleader make any error concerning the ECPR guidelines and protocols (cfr lesson given)?***

|  | | *Control* | *Intervention* |
| --- | --- | --- | --- |
| PRE | n/N (%) | 0/ 0 | 0/ 0 |
| POST | n/N (%) | 0/ 0 | 3/ 39 ( 7.69%) |
|  | | | |

### ***Timing of activation ECPR protocol***

x

|  | | | *95% confidence interval* | |  |
| --- | --- | --- | --- | --- | --- |
|  | *Minutes* | *Completed Performed* | *Lower Limit* | *Upper Limit* |  |
|  | 10 | 92.50 | 90.06 | 94.53 |  |
|  | 15 | 97.50 | 96.65 | 98.18 |  |
| The confidence interval for the Kaplan-Meier estimates is based on the log-log transformation, using the Greenwood formula for the standard error | | | | | |

|  | | | *95% confidence interval* | |  |
| --- | --- | --- | --- | --- | --- |
|  | *Percentile* | *Minutes* | *Lower Limit* | *Upper Limit* |  |
|  | 25 | 6.52 | 5.33 | 7.05 |  |
|  | 50 | 7.50 | 6.83 | 8.45 |  |
|  | 75 | 8.96 | 8.08 | 9.65 |  |
| The confidence interval is based on the log-log transformation | | | | | |
